# Supplementary figures and images for: The impact of short-term machine perfusion on the risk of cancer recurrence after rat liver transplantation with donors after circulatory death
Source: PLoS One. 2019 Nov 25;14(11):e0224890. doi: 10.1371/journal.pone.0224890 (PMC6876876; doi:10.1371/journal.pone.0224890)

**
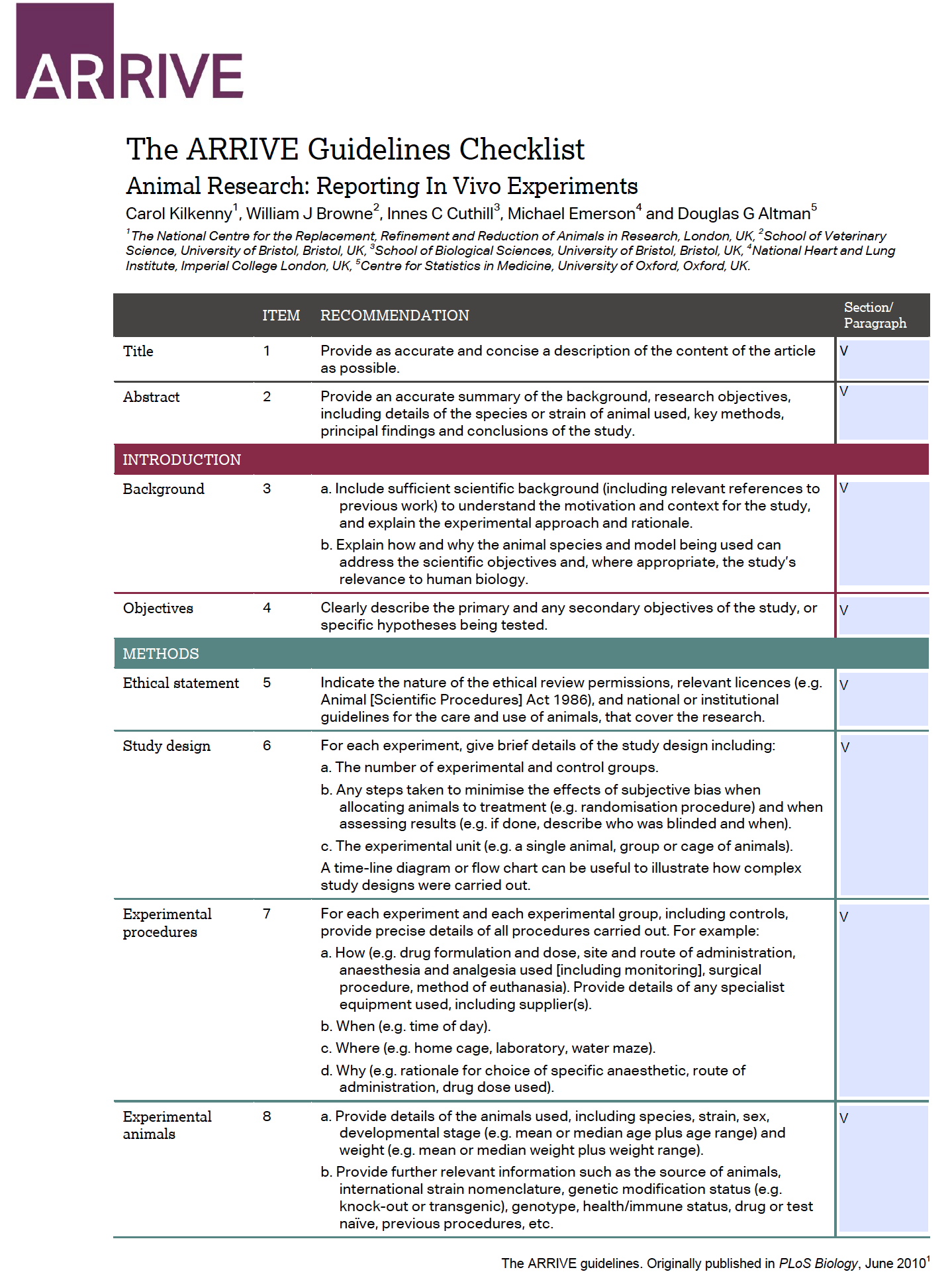
**


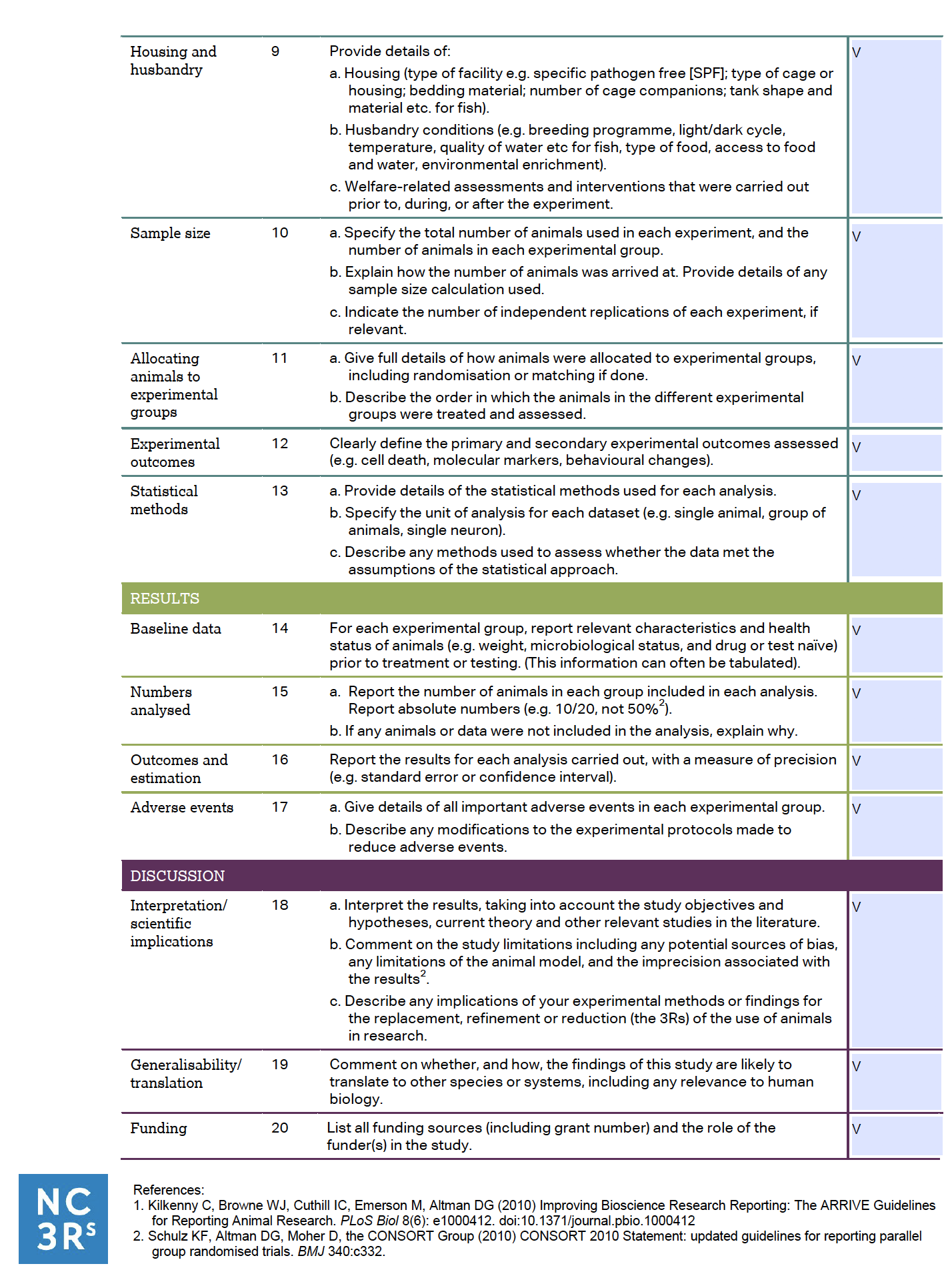

Supplement: S1 Checklist — (DOCX) [file pone.0224890.s001.docx]

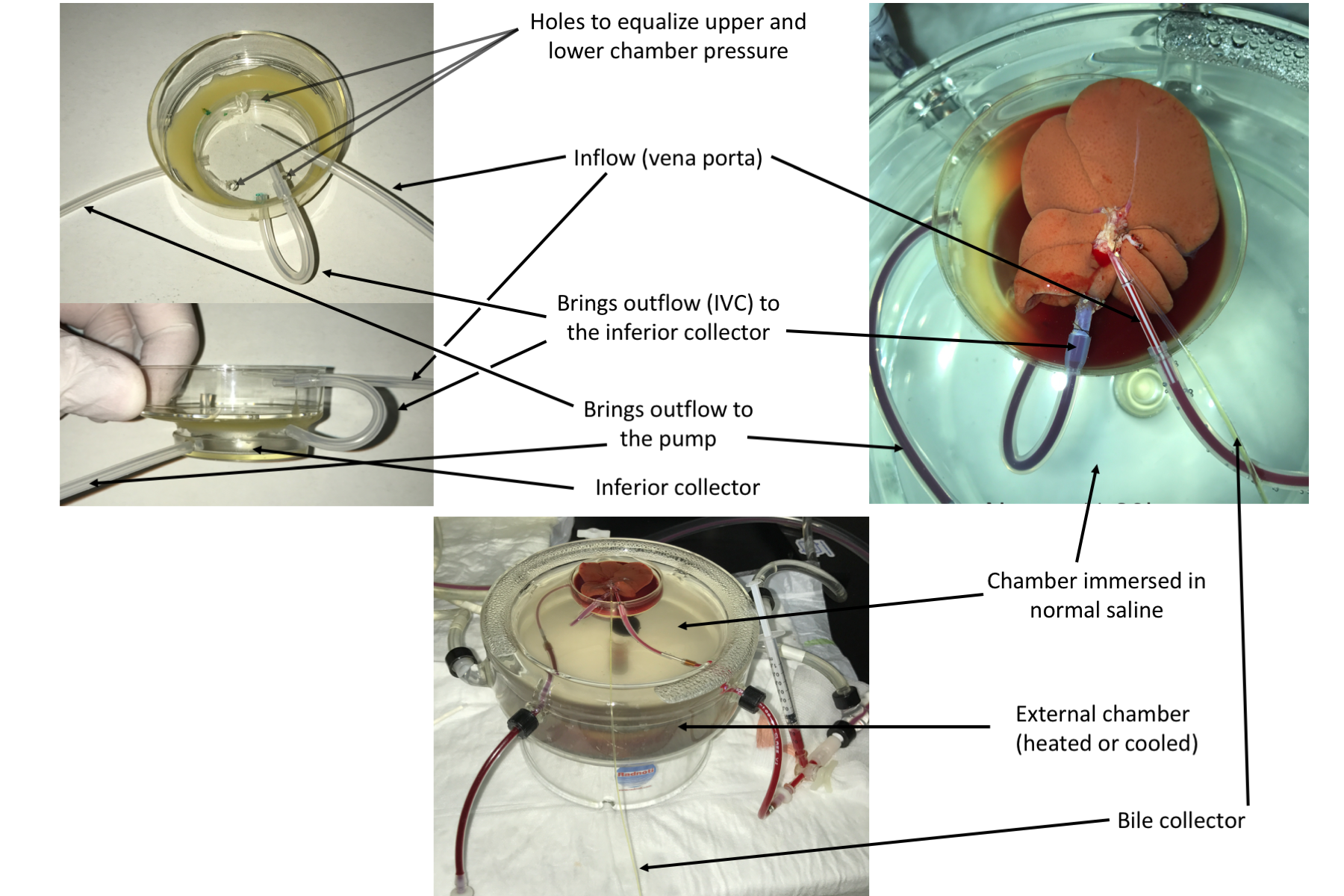

Supplement: S1 Fig — The perfusion chamber is a semi-open system composed of a lower chamber (35x10mm petri dish) glued to the inferior surface of a larger open chamber (petri dish 60x15mm). The two chambers communicate through three 2mm holes in the floor of the upper chamber. Chambers are filled with cold solution and liver graft lays into the upper chamber (the SHVC being previously tied). The vena porta (inflow) and the IVC (outflow) are connected to cannulas fitting their size without excessive stretching. The IVC cannula is connected to a soft silicon tubing linking the upper and the lower chamber. The perfusion solution runs through the graft and is collected into the lower chamber without contaminating the fluid in the upper chamber. The solution collecting into the lower chamber goes to the pump through a single silicon tubing. Of note, the cyclic pressure fluctuations (typical of peristaltic pumps) potentially causing reduced aspiration in the lower chamber are compensated by the holes interconnecting the two chambers. IVC = infrahepatic vena cava. (TIFF) [file pone.0224890.s002.tiff]
